# Association of hospital-arrival rhythm and ROSC with outcomes after ECPR for OHCA with initial shockable rhythm

**DOI:** 10.1186/s13054-025-05698-9

**Published:** 2025-11-03

**Authors:** Kenji Kandori, Tasuku Matsuyama, Tetsuhisa Kitamura, Hiromichi Narumiya, Wataru Ishii, Masahito Hitosugi, Yohei Okada

**Affiliations:** 1 Department of Emergency and Critical Care Medicine, Japanese Red Cross Society, Kyoto Daini Hospital, 355-5 Haruobi-cho Kamigyo-ku, Kyoto, 602–8026 Japan; 2 Department of Legal Medicine, Shiga University of Medical Science, Tsukinowa, Seta, Otsu, Shiga, 520–2192 Japan; 3 Department of Emergency Medicine, Kyoto Prefectural University of Medicine, Kaji-cho 465, Kamigyo-ku, Kyoto, 602–8566 Japan; 4 Division of Environmental Medicine and Population Sciences, Department of Social and Environmental Medicine, Graduate School of Medicine, Osaka University, 2–2, Yamada-oka, Suita, Osaka, 565–0871 Japan; 5 Department of Preventive Services, Graduate school of medicine, Kyoto University, Kyoto, 606–8501 Yoshida-Konoe-cho, Sakyo-ku Japan; 6 Health Services and Systems Research, Duke-NUS Medical School, National University of Singapore, 8 College Road, Singapore, 169857 Singapore

## Abstract

**Supplementary Information:**

The online version contains supplementary material available at 10.1186/s13054-025-05698-9.

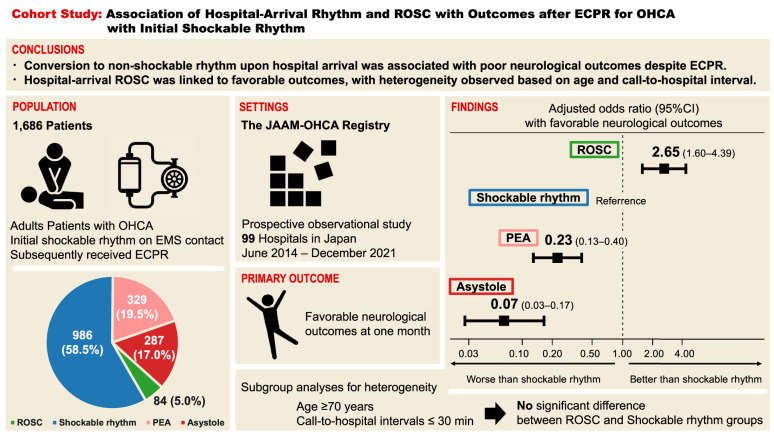

Out-of-hospital cardiac arrest (OHCA) presenting with initial shockable rhythm generally has a relatively favorable prognosis, making these patients suitable candidates for extracorporeal cardiopulmonary resuscitation (ECPR) [1]. Given ECPR’s invasiveness and resource intensity, precise candidate selection is critical. Current eligibility criteria primarily rely on prehospital factors—initial rhythm, age, witnessed arrest, bystander CPR, and call-to-hospital interval—which may not fully capture the patient’s physiological status at ECPR initiation, as the condition can change dynamically during resuscitation. We aimed to evaluate whether hospital-arrival cardiac rhythm and return of spontaneous circulation (ROSC), hypothesized to better reflect pre-ECPR physiological status, are associated with neurological outcomes, and to explore outcome heterogeneity in ECPR recipients after OHCA with initial shockable rhythm.

We conducted a secondary analysis of the Japanese Association for Acute Medicine OHCA (JAAM-OHCA) registry, a nationwide prospective multicenter database (June 2014–December 2021) [2]. Adult patients (≥ 18 years) with medical-cause OHCA, initial shockable rhythm on emergency medical service contact, and subsequent ECPR were included. Arrival status was categorized as: (1) ROSC, (2) persistent shockable rhythm, (3) pulseless electrical activity (PEA), or (4) asystole. The primary outcome was 1-month favorable neurological status (Cerebral Performance Category 1–2); the secondary outcome was 1-month survival. Adjusted odds ratios (aORs) for favorable neurological outcome were calculated using multiple logistic regression. Subgroup analyses by age and call-to-hospital interval assessed heterogeneity.

Of 81,234 registry patients, 1,686 met inclusion criteria (Supplemental Fig. 1). Median age was 60 years (IQR, 49–69), and 86.2% were men (Supplemental Table 1). Upon hospital arrival, 5.0% had achieved ROSC, 58.5% remained in shockable rhythm, and 36.5% converted to non-shockable rhythms (PEA, 19.5%; asystole, 17.0%). Overall, 13.2% achieved favorable neurological outcomes. Rates by group were: 36.9% (ROSC), 17.3% (shockable), 4.6% (PEA), and 1.7% (asystole) (Supplemental Table 2).

Using persistent shockable rhythm as the reference, aORs (95% confidence intervals) for favorable neurological outcome were 2.65 (1.60–4.39) for ROSC, 0.23 (0.13–0.40) for PEA, and 0.07 (0.03–0.17) for asystole (Fig. 1 and Supplemental Table 3). Supplemental Table 4 shows analysis results for 1-month survival.

**Fig. 1:**
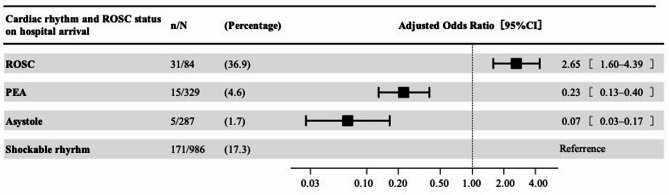
Forest plot of adjusted associations of hospital-arrival cardiac rhythm and rosc status with 1-month neurological outcomes. Adjusted associations of cardiac rhythm and ROSC status upon hospital arrival with 1-Month favorable neurological outcomes in patients with OHCA who presented with initial shockable rhythm and subsequently received ECPR. CI, confidence interval; ECPR, extracorporeal cardiopulmonary resuscitation; OHCA, out-of-hospital cardiac arrest; PEA, pulseless electrical activity; ROSC, return of spontaneous circulation

Subgroup analyses demonstrated that PEA and asystole were associated with poor neurological outcomes across nearly all subgroups (Supplemental Fig. 2, 3 and Supplemental Tables 5, 6). Notably, among patients aged ≥ 70 years and those with call-to-hospital intervals ≤ 30 min, no significant difference in outcomes was detected between the ROSC and shockable rhythm groups.

These results highlight the significant association between hospital-arrival cardiac rhythm and ROSC status, and neurological outcomes after ECPR in OHCA patients with initial shockable rhythm.

Conversion from shockable to non-shockable rhythm upon hospital arrival likely reflects progression into the “metabolic phase” in the three-phase model, in which ischemia–reperfusion injury leads to irreversible neurological damage [3, 4]. This may explain the.

uniformly poor outcomes regardless of age or call-to-hospital interval. While ROSC upon arrival was generally associated with better outcomes, this benefit was attenuated in elderly patients (≥ 70 years) and those with short call-to-hospital intervals (≤ 30 min). This suggests that ROSC in older patients might not indicate favorable recovery of physiology, possibly reflecting transient, low-quality circulatory recovery or preexisting comorbidities limiting reversibility [5]. Conversely, in patients with shorter call-to-hospital intervals, persistent shockable rhythm might indicate better myocardial viability and less ischemia–reperfusion injury, preserving neurological potential and resulting in outcomes comparable to patients with ROSC.

Our findings suggest that incorporating hospital-arrival cardiac rhythm and ROSC status—objective and rapidly available measures—into existing ECPR selection frameworks (which typically consider age, witnessed arrest, bystander CPR, and low-flow time) could markedly improve prognostic precision [1]. Patients who convert to non-shockable rhythms upon arrival exhibit consistently poor neurological outcomes, even when young or rapidly transported, and might merit exclusion or careful deferment of ECPR. Conversely, both ROSC and persistent shockable rhythm identify candidates with potential benefit; however, their prognostic value is affected by age (notably ≥ 70 years) and call‑to‑hospital interval (≤ 30 min), underscoring the need for dynamic thresholds rather than rigid cut-offs. Therefore, we propose the development of a dynamic, multidimensional, interaction-aware eligibility scoring system that integrates hospital-arrival cardiac rhythm, ROSC status, and their interactions with age and call-to-hospital interval into existing ECPR eligibility criteria [1]. Prospective validation of such a model could optimize ECPR resource allocation and improve patient outcomes by enabling real‑time individualized assessments. Future research should validate this approach across diverse populations and healthcare settings to confirm its generalizability and impact.

Hospital‑arrival cardiac rhythm and ROSC status are significantly associated with neurological outcomes in patients with OHCA presenting with initial shockable rhythm who received ECPR. Conversion to non-shockable rhythm is linked to poor outcomes, whereas achieving ROSC predicts favorable outcomes. These associations vary according to patient age and call-to-hospital interval, highlighting the need to develop more detailed and personalized ECPR eligibility criteria.

## Supplementary Information

Supplementary Material 1

Supplementary Material 2

Supplementary Material 3

## Data Availability

The JAAM-OHCA Registry data are not publicly available because the ethics committee does not permit it.

## Abbreviations

aORs: Adjusted odds ratios
ECPR: Extracorporeal cardiopulmonary resuscitation
OHCA: Out-of-hospital cardiac arrest
PEA: Pulseless electrical activity
ROSC: Return of spontaneous circulation
