# Supplementary material for: Association of hospital-arrival rhythm and ROSC with outcomes after ECPR for OHCA with initial shockable rhythm
Source: Crit Care. 2025 Nov 3;29:466. doi: 10.1186/s13054-025-05698-9 (PMC12581255; doi:10.1186/s13054-025-05698-9)
Supplement: Supplementary file 1 — Supplementary Material 1 [file 13054_2025_5698_MOESM1_ESM.pdf]

Supplemental Figures and Tables

**Supplemental Figure 1**	Study flowchart.
**Supplemental Figure 2**	Age-Stratified Forest Plot of Adjusted Associations of Hospital-Arrival Cardiac Rhythm and ROSC Status With 1-Month Neurological Outcomes.
**Supplemental Figure 3**	Call-to-Hospital Interval-Stratified Forest Plot of Adjusted Associations of Hospital-Arrival Cardiac Rhythm and ROSC Status With 1-Month Neurological Outcomes.
**Supplemental Table 1**	Demographics and Characteristics Based on Cardiac Rhythm and ROSC Status upon Hospital Arrival
**Supplemental Table 2**	Outcomes Based on Cardiac Rhythm and ROSC Status upon Hospital Arrival
**Supplemental Table 3**	Associations of Cardiac Rhythm and ROSC Status upon Hospital Arrival With 1-Month Favorable Neurological Outcomes in Patients With OHCA Presented With Initial Shockable Rhythm and Subsequently Received ECPR
**Supplemental Table 4**	Associations of Cardiac Rhythm and ROSC Status on Hospital Arrival With 1-Month Survival in Patients With OHCA Presented With Initial Shockable Rhythm and Subsequently Received ECPR
**Supplemental Table 5**	Age-Stratified Subgroup Analyses of the Association Between Hospital-Arrival Cardiac Rhythm and ROSC Status and 1-Month Favorable Neurological Outcomes in Patients With OHCA Presented With Initial Shockable Rhythm and Subsequently Received ECPR
**Supplemental Table 6**	Call-to-Hospital Interval-Stratified Subgroup Analyses of the Association Between Hospital-Arrival Cardiac Rhythm and ROSC Status and 1-Month Favorable Neurological Outcomes in Patients With OHCA Presented With Initial Shockable Rhythm and Subsequently Received ECPR

Supplemental Figure 1. Study flowchart.

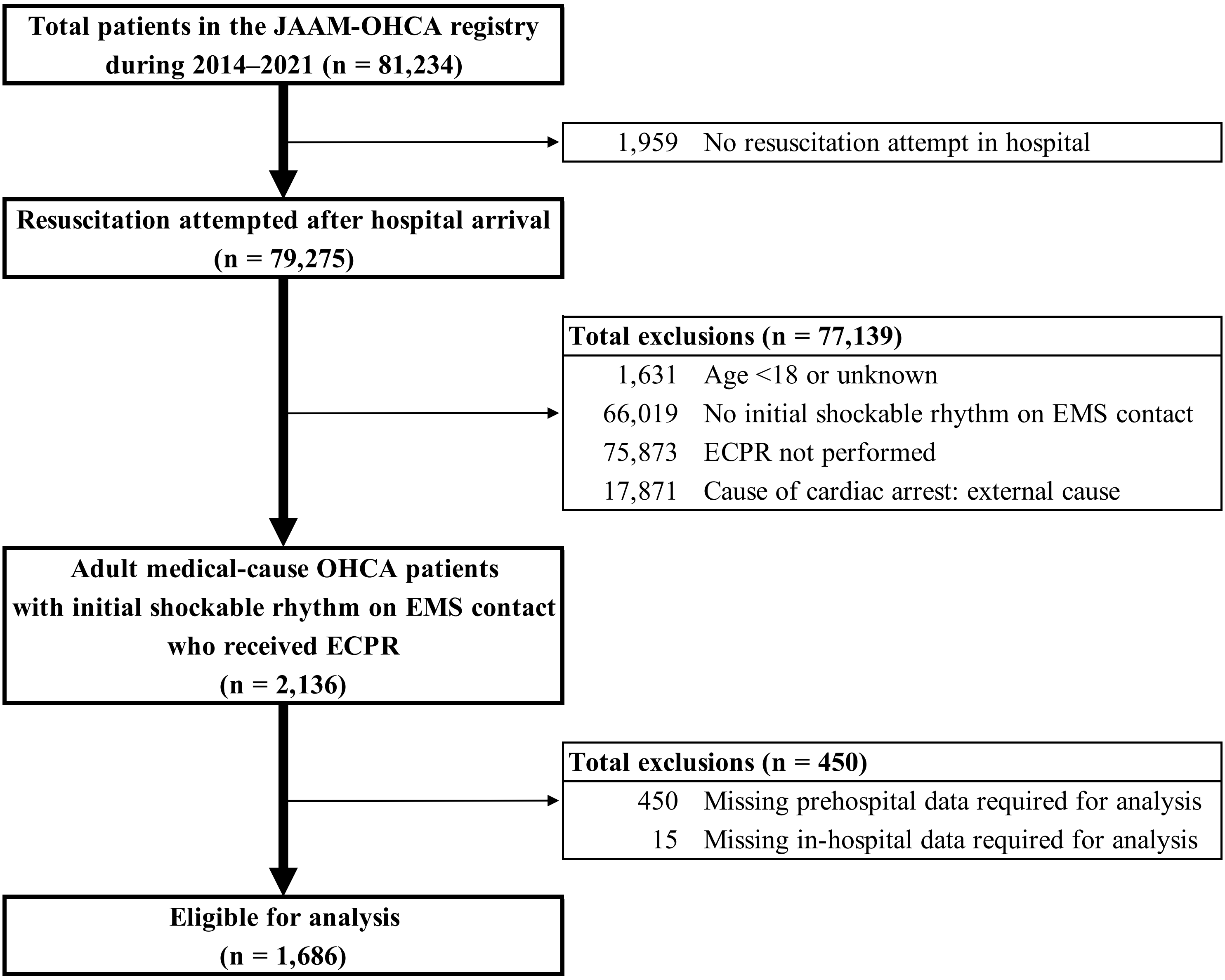

ECPR, extracorporeal cardiopulmonary resuscitation; EMS, emergency medical service; JAAM-OHCA, Japanese Association for Acute Medicine Out-of-Hospital Cardiac Arrest.

Supplemental Figure 2. Age-Stratified Forest Plot of Adjusted Associations of Hospital-Arrival Cardiac Rhythm and ROSC Status With 1-Month Neurological Outcomes.

Age subgroup	n/N	(%)	Adjusted Odds Ratio ［95%CI］
**ROSC**			
**Overall**	31/84	(36.9)	2.65 ［ 1.60–4.39 ］
**18–59 years**	25/42	(59.5)	4.91 ［ 2.45–9.84 ］
**60–69 years**	5/17	(29.4)	2.17 ［ 0.70–6.76 ］
**70 years–**	1/25	(4.0)	0.22 ［ 0.03–1.88 ］
**PEA**			
**Overall**	15/329	(4.6)	0.23 ［ 0.13–0.40 ］
**18–59 years**	9/151	(6.0)	0.25 ［ 0.12–0.50 ］
**60–69 years**	4/110	(3.6)	0.19 ［ 0.07–0.56 ］
**70 years–**	2/68	(2.9)	0.20 ［ 0.04–0.90 ］
**Asystole**			
**Overall**	5/287	(1.7)	0.07 ［ 0.03–0.17 ］
**18–59 years**	4/177	(2.3)	0.08 ［ 0.03–0.21 ］
**60–69 years**	0/55	(0.0)	0.00 ［ 0.00– ］
**70 years–**	1/55	(1.8)	0.11 ［ 0.01–0.90 ］

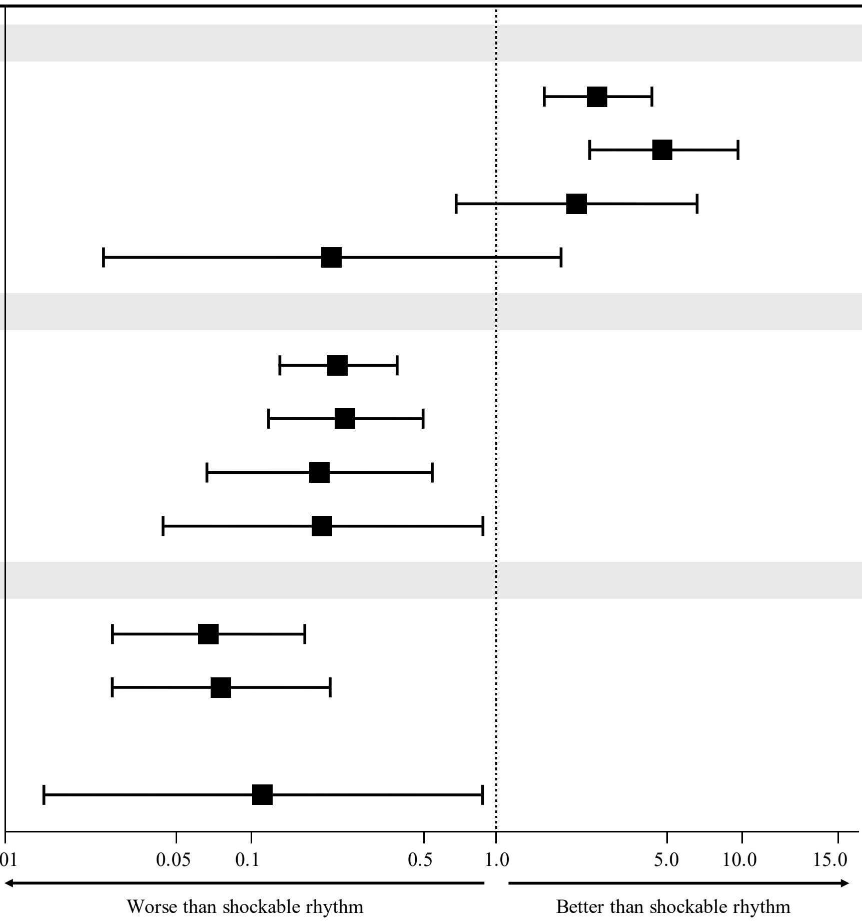

Age-stratified adjusted odds ratios for 1-Month favorable neurological outcomes by cardiac rhythm and ROSC status in patients with OHCA who presented with initial shockable rhythm and subsequently received ECPR.

CI, confidence interval; ECPR, extracorporeal cardiopulmonary resuscitation; OHCA, out-of-hospital cardiac arrest; PEA, pulseless electrical activity; ROSC, return of spontaneous circulation.

Supplemental Figure 3. Call-to-Hospital Interval-Stratified Forest Plot of Adjusted Associations of Hospital-Arrival Cardiac Rhythm and ROSC Status With 1-Month Neurological Outcomes.

Time subgroup	n/N	(%)	Adjusted Odds Ratio［95%CI］
**ROSC**			
**Overall**	31/84	(36.9)	2.65 ［ 1.60–4.39 ］
**0–20 min**	5/16	(31.3)	1.83 ［ 0.57–5.84 ］
**21–30 min**	13/35	(37.1)	2.10 ［ 0.97–4.53 ］
**31–40 min**	5/18	(27.8)	4.20 ［ 1.23–14.26 ］
**41 min–**	8/15	(53.3)	5.67 ［ 1.24–25.94 ］
**PEA**			
**Overall**	15/329	(4.6)	0.23 ［ 0.13–0.40 ］
**0–20 min**	2/66	(3.0)	0.12 ［ 0.03–0.53 ］
**21–30 min**	8/137	(5.8)	0.24 ［ 0.11–0.52 ］
**31–40 min**	5/95	(5.3)	0.44 ［ 0.16–1.22 ］
**41 min–**	0/31	(0.0)	0.00 ［ 0.00– ］
**Asystole**			
**Overall**	5/287	(1.7)	0.07 ［ 0.03–0.17 ］
**0–20 min**	2/55	(3.6)	0.14 ［ 0.03–0.60 ］
**21–30 min**	2/132	(1.5)	0.05 ［ 0.01–0.21 ］
**31–40 min**	0/68	(0.0)	0.00 ［ 0.00– ］
**41 min–**	1/32	(3.1)	0.06 ［ 0.01–0.66 ］

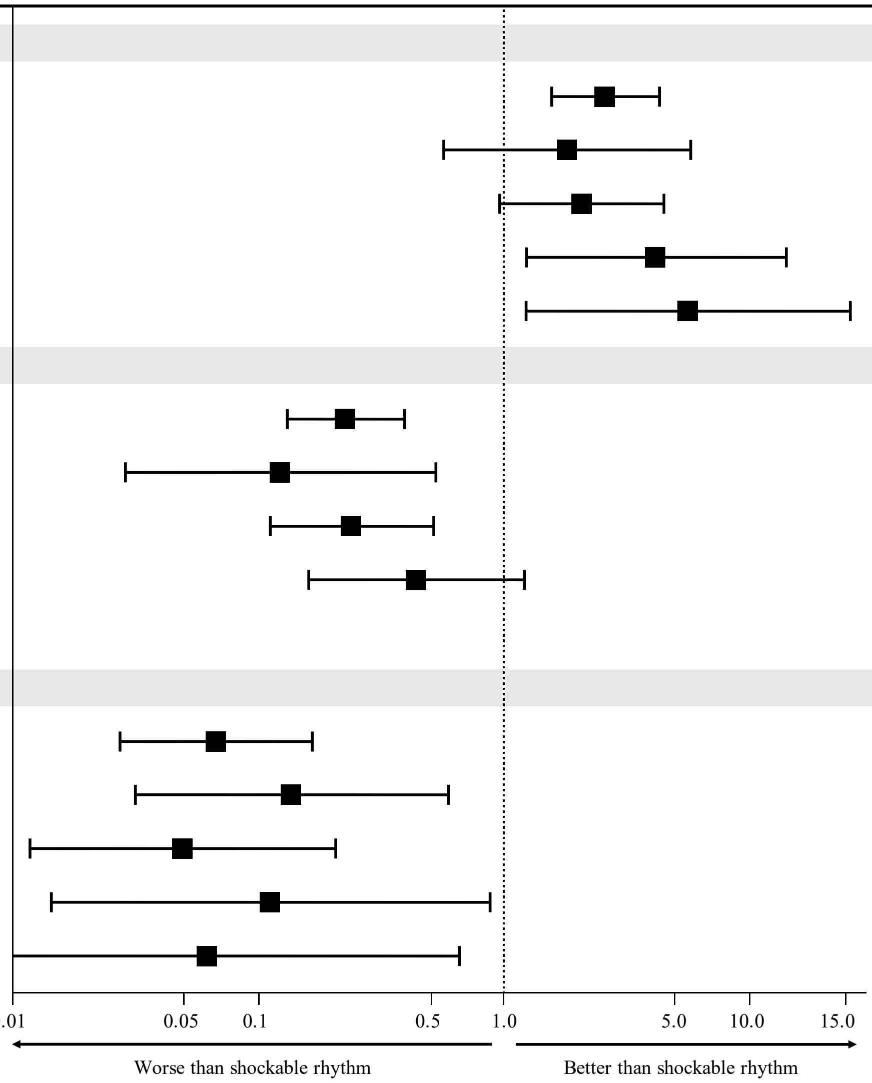

Call-to-hospital interval-stratified forest plot of adjusted ratios for 1-Month favorable neurological outcomes by cardiac rhythm and ROSC status upon hospital arrival in patients with OHCA who presented with initial shockable rhythm and subsequently received ECPR.

CI, confidence interval; ECPR, extracorporeal cardiopulmonary resuscitation; OHCA, out-of-hospital cardiac arrest; PEA, pulseless electrical activity; ROSC, return of spontaneous circulation.

Supplemental Table 1. Demographics and Characteristics Based on Cardiac Rhythm and ROSC Status upon Hospital Arrival

Variables	Overall (N = 1,686)	ROSC (N = 84)	Shockable rhythm (N = 986)	PEA (N = 329)	Asystole (N = 287)
**Age, y**	60 [49–69]	59.5 [50–72]	61 [50–69]	61 [50–69]	56 [46–66]
**Age group, y**					
18–59	824 (48.9 %)	42 (50.0 %)	454 (46.0 %)	151 (45.9 %)	177 (61.7 %)
60–69	489 (29.0 %)	17 (20.2 %)	307 (31.1 %)	110 (33.4 %)	55 (19.2 %)
≥70	373 (22.1 %)	25 (29.8 %)	225 (22.8 %)	68 (20.7 %)	55 (19.2 %)
**Male sex**	1,453 (86.2 %)	73 (86.9 %)	862 (87.4 %)	281 (85.4 %)	237 (82.6 %)
**Cause of cardiac arrest**					
Cardiac disease	1,604 (95.1 %)	80 (95.2 %)	948 (96.1 %)	302 (91.8 %)	274 (95.5 %)
Cerebrovascular disease	9 (0.5 %)	0 (0.0 %)	7 (0.7 %)	2 (0.6 %)	0 (0.0 %)
Respiratory disease	1 (0.1 %)	0 (0.0 %)	1 (0.1 %)	0 (0.0 %)	0 (0.0 %)
Malignant tumor	1 (0.1 %)	0 (0.0 %)	0 (0.0 %)	0 (0.0 %)	1 (0.3 %)
Others or unknown	71 (4.2 %)	4 (4.8 %)	30 (3.0 %)	25 (7.6 %)	12 (4.2 %)
**Prehospital information**					
**Witnessed cardiac arrest, n**	1,331 (78.9 %)	67 (79.8 %)	783 (79.4 %)	261 (79.3 %)	220 (76.7 %)
**Bystander CPR, n**	934 (55.4 %)	52 (61.9 %)	557 (56.5 %)	170 (51.7 %)	155 (54.0 %)
**Bystander defibrillation, n**	152 (9.0 %)	7 (8.3 %)	101 (10.2 %)	21 (6.4 %)	23 (8.0 %)
**Prehospital adrenaline administration, n**	674 (40.0 %)	26 (31.0 %)	440 (44.6 %)	128 (38.9 %)	80 (27.9 %)
**Prehospital advanced airway management, n**	962 (57.1 %)	44 (52.4 %)	542 (55.0 %)	210 (63.8 %)	166 (57.8 %)
**Call-to-hospital interval, min**	27 [21–34]	27 [23–38]	27 [20–33]	28 [21–34]	28 [23–34]
0–20	401 (23.8 %)	16 (19.0 %)	264 (26.8 %)	66 (20.1 %)	55 (19.2 %)
21–30	688 (40.8 %)	35 (41.7 %)	384 (38.9 %)	137 (41.6 %)	132 (46.0 %)
31–40	419 (24.9 %)	18 (21.4 %)	238 (24.1 %)	95 (28.9 %)	68 (23.7 %)
≥41	178 (10.6 %)	15 (17.9 %)	100 (10.1 %)	31 (9.4 %)	32 (11.1 %)
**In-hospital information**					
**Intervention, n**					
IABP	1,056 (62.6 %)	21 (25.0 %)	344 (34.9 %)	124 (37.7 %)	141 (49.1 %)
CAG	1,332 (79.0 %)	78 (92.9 %)	791 (80.2 %)	264 (80.2 %)	199 (69.3 %)
PCI	763 (45.3 %)	51 (60.7 %)	465 (47.2 %)	155 (47.1 %)	92 (32.1 %)
TTM	826 (49.0 %)	43 (51.2 %)	497 (50.4 %)	170 (51.7 %)	116 (40.4 %)
**Call-to-ECMO, min**	56 [47–69]	73 [56–141]	55 [46–66]	56 [48–69]	58 [49–71]
**Hospitalization, n**	1,513 (89.7 %)	79 (94.0 %)	897 (91.0 %)	297 (90.3 %)	240 (83.6 %)

Values are presented as medians (interquartile range) or numbers (percentage).
CAG, coronary angiography; CPR, cardiopulmonary resuscitation; ECMO, extracorporeal membrane oxygenation; IABP, intra-aortic balloon pumping; PCI, percutaneous coronary intervention; PEA, pulseless electrical activity; ROSC, return of spontaneous circulation; TTM, targeted temperature management.

Supplemental Table 2. Outcomes Based on Cardiac Rhythm and ROSC Status upon Hospital Arrival

Variables	Overall (N = 1,686)	ROSC (N = 84)	Shockable rhythm (N = 986)	PEA (N = 329)	Asystole (N = 287)
**One-month survival**	477 (28.3 %)	45 (53.6 %)	322 (32.7 %)	78 (23.7 %)	32 (11.1 %)
**One-month favorable neurological outcome**	222 (13.2 %)	31 (36.9 %)	171 (17.3 %)	15 (4.6 %)	5 (1.7 %)

Values are presented as numbers (percentages).
PEA, pulseless electrical activity; ROSC, return of spontaneous circulation.

Supplemental Table 3. Associations of Cardiac Rhythm and ROSC Status upon Hospital Arrival With 1-Month Favorable Neurological Outcomes in Patients With OHCA Presented With Initial Shockable Rhythm and Subsequently Received ECPR

	n/N	Percentage	Crude OR	95% CI	Adjusted OR	95% CI
**Cardiac rhythm and ROSC status on hospital arrival**						
**Shockable rhythm**	171/986	(17.3)	Reference		Reference	
**ROSC**	31/84	(36.9)	2.79	(1.74–4.47)	2.65	(1.60–4.39)
**PEA**	15/329	(4.6)	0.23	(0.13–0.39)	0.23	(0.13–0.40)
**Asystole**	5/287	(1.7)	0.08	(0.03–0.21)	0.07	(0.03–0.17)
**Age group, y**						
**18–59**	139/824	(16.9)	Reference		Reference	
**60–69**	53/489	(10.8)	0.60	(0.43–0.84)	0.56	(0.39–0.79)
**70–**	30/373	(8.0)	0.43	(0.28–0.65)	0.39	(0.25–0.60)
**Sex**						
**Male**	191/1453	(13.2)	0.99	(0.66–1.48)	0.82	(0.53–1.28)
**Witnessed cardiac arrest**						
**Yes**	186/1,331	(14.0)	1.44	(0.99–2.10)	1.33	(0.89–1.98)
**Bystander CPR**						
**Yes**	139/934	(14.9)	1.41	(1.05–1.88)	1.25	(0.90–1.73)
**Bystander defibrillation**						
**Yes**	32/152	(21.1)	1.89	(1.24–2.87)	1.40	(0.87–2.25)
**Prehospital advanced airway management**						
**Yes**	91/962	(9.5)	0.47	(0.35–0.63)	0.63	(0.45–0.87)
**Prehospital adrenaline administration**						
**Yes**	61/674	(9.1)	0.53	(0.38–0.72)	0.55	(0.39–0.78)
**Call-to-hospital interval, min**						
**0–20**	63/401	(15.7)	Reference		Reference	
**21–30**	99/688	(13.3)	0.90	(0.64–1.27)	1.07	(0.74–1.55)
**31–40**	35/419	(8.4)	0.49	(0.32–0.76)	0.67	(0.41–1.07)
**41–**	25/178	(14.0)	0.88	(0.53–1.45)	1.11	(0.64–1.94)

Adjusted for confounding variables, including cardiac rhythm and ROSC status on hospital arrival, patient age, sex, presence of witnessed arrest, bystander CPR, bystander defibrillation, prehospital advanced airway management, prehospital adrenaline administration, and call-to-hospital arrival interval. Age and call-to-hospital arrival interval were categorized based on quartiles and adjusted to round numbers for easier clinical application.

CI, confidence interval; CPR, cardiopulmonary resuscitation; ECPR, extracorporeal cardiopulmonary resuscitation; OHCA, out-of-hospital cardiac arrest; OR, odds ratio; PEA, pulseless electrical activity; and ROSC, return of spontaneous circulation.

Supplemental Table 4. Associations of Cardiac Rhythm and ROSC Status on Hospital Arrival With 1-Month Survival in Patients With OHCA Presented With Initial Shockable Rhythm and Subsequently Received ECPR

	n/N	Percentage	Crude OR	95% CI	Adjusted OR	95% CI
**Cardiac rhythm and ROSC status on hospital arrival**						
**Shockable rhythm**	322/986	(32.7)	Reference		Reference	
**ROSC**	45/84	(53.6)	2.38	(1.52–3.73)	2.56	(1.61–4.09)
**PEA**	78/329	(23.7)	0.64	(0.48–0.85)	0.65	(0.48–0.87)
**Asystole**	32/287	(11.2)	0.26	(0.18–0.38)	0.24	(0.16–0.35)
**Age group, y**						
**18–59**	258/824	(31.3)	Reference		Reference	
**60–69**	139/489	(28.4)	0.87	(0.68–1.11)	0.80	(0.62–1.03)
**70–**	80/373	(21.5)	0.60	(0.45–0.80)	0.54	(0.40–0.73)
**Sex**						
**Male**	414/1,453	(28.5)	1.08	(0.79–1.47)	0.96	(0.69–1.33)
**Witnessed cardiac arrest**						
**Yes**	384/1,331	(28.9)	1.14	(0.88–1.49)	1.14	(0.86–1.50)
**Bystander CPR**						
**Yes**	270/934	(28.9)	1.07	(0.86–1.33)	0.98	(0.78–1.24)
**Bystander defibrillation**						
**Yes**	52/152	(34.2)	1.36	(0.95–1.93)	1.22	(0.83–1.81)
**Prehospital advanced airway management**						
**Yes**	245/962	(25.5)	0.72	(0.59–0.90)	0.91	(0.72–1.16)
**Prehospital adrenaline administration**						
**Yes**	166/674	(24.6)	0.74	(0.59–0.92)	0.79	(0.62–1.00)
**Call-to-hospital interval, min**						
**0–20**	145/401	(36.2)	Reference		Reference	
**21–30**	210/688	(30.5)	0.78	(0.60–1.01)	0.83	(0.63–1.09)
**31–40**	86/419	(20.5)	0.46	(0.33–0.62)	0.50	(0.36–0.70)
**41–**	36/178	(20.2)	0.45	(0.29–0.68)	0.46	(0.29–0.71)

Adjusted for confounding variables, including cardiac rhythm and ROSC status on hospital arrival, patient age, sex, presence of witnessed arrest, bystander CPR, bystander defibrillation, prehospital advanced airway management, prehospital adrenaline administration, and call-to-hospital arrival interval. Age and call-to-hospital arrival interval were categorized based on quartiles and adjusted to round numbers for easier clinical application.

CI, confidence interval; CPR, cardiopulmonary resuscitation; ECPR, extracorporeal cardiopulmonary resuscitation; OHCA, out-of-hospitalcardiac arrest; OR, odds ratio; PEA, pulseless electrical activity; and ROSC, return of spontaneous circulation.

Supplemental Table 5. Age-Stratified Subgroup Analyses of the Association Between Hospital-Arrival Cardiac Rhythm and ROSC Status and 1-Month Favorable Neurological Outcomes in Patients With OHCA Presented With Initial Shockable Rhythm and Subsequently Received ECPR

	n/N	(%)	Crude OR	(95% CI)	Adjusted OR	(95% CI)
**Age group: 18–59 years**						
**Shockable rhythm**	101/454	(22.3)	Reference		Reference	
**ROSC**	25/42	(59.5)	5.14	(2.69–10.05)	4.91	(2.45–9.84)
**PEA**	9/151	(6.0)	0.22	(0.10–0.43)	0.25	(0.12–0.50)
**Asystole**	4/177	(2.3)	0.08	(0.02–0.20)	0.08	(0.03–0.21)
**Age group: 60–69 years**						
**Shockable rhythm**	44/307	(14.3)	Reference		Reference	
**ROSC**	5/17	(29.4)	2.49	(0.76–7.08)	2.17	(0.70–6.76)
**PEA**	4/110	(3.6)	0.23	(0.07–0.57)	0.19	(0.07–0.56)
**Asystole**	0/55	(0.0)	0.00	(–0.22)	0.00	(0.00–)
**Age group: 70 years–**						
**Shockable rhythm**	26/225	(11.6)	Reference		Reference	
**ROSC**	1/25	(4.0)	0.32	(0.02–1.61)	0.22	(0.03–1.88)
**PEA**	2/68	(2.9)	0.23	(0.04–0.81)	0.20	(0.04–0.90)
**Asystole**	26/225	(1.8)	0.14	(0.01–0.69)	0.11	(0.01–0.90)

Adjusted for confounding variables, including cardiac rhythm and ROSC status on hospital arrival, patient sex, presence of witnessed arrest, bystander CPR, bystander defibrillation, prehospital advanced airway management, prehospital adrenaline administration, and call-to-hospital arrival interval. Call-to-hospital arrival interval was categorized based on quartiles and adjusted to round numbers for easier clinical application.

CI, confidence interval; CPR, cardiopulmonary resuscitation; ECPR, extracorporeal cardiopulmonary resuscitation; OHCA, out-of-hospitalcardiac arrest; OR, odds ratio; PEA, pulseless electrical activity; and ROSC, return of spontaneous circulation.

Supplemental Table 6. Call-to-Hospital Interval-Stratified Subgroup Analyses of the Association Between Hospital-Arrival Cardiac Rhythm and ROSC Status and 1-Month Favorable Neurological Outcomes in Patients With OHCA Presented With Initial Shockable Rhythm and Subsequently Received ECPR

	n/N	(%)	Crude OR	(95% CI)	Adjusted OR	(95% CI)
**Call-to-hospital interval: 0–20 min**						
**Shockable rhythm**	54/264	(20.5)	Reference		Reference	
**ROSC**	5/16	(31.3)	1.77	(0.54–5.08)	1.83	(0.57–5.84)
**PEA**	2/66	(3.0)	0.12	(0.02–0.41)	0.12	(0.03–0.53)
**Asystole**	2/55	(3.6)	0.15	(0.02–0.49)	0.14	(0.03–0.60)
**Call-to-hospital interval: 21–30 min**						
**Shockable rhythm**	76/384	(19.8)	Reference		Reference	
**ROSC**	13/35	(37.1)	2.39	(1.13–4.91)	2.10	(0.97–4.53)
**PEA**	8/137	(5.8)	0.25	(0.11–0.51)	0.24	(0.11–0.52)
**Asystole**	2/132	(1.5)	0.06	(0.01–0.20)	0.05	(0.01–0.21)
**Call-to-hospital interval: 31–40 min**						
**Shockable rhythm**	25/238	(10.5)	Reference		Reference	
**ROSC**	5/18	(27.8)	3.28	(0.99–9.52)	4.20	(1.23–14.26)
**PEA**	5/95	(5.3)	0.47	(0.16–1.18)	0.44	(0.16–1.22)
**Asystole**	0/68	(0.0)	0.00	(–0.25)	0.00	(0.00–)
**Call-to-hospital interval: 41 min–**						
**Shockable rhythm**	16/100	(16.0)	Reference		Reference	
**ROSC**	8/15	(53.3)	6.00	(1.91–19.49)	5.67	(1.24–25.94)
**PEA**	0/31	(0.0)	0.00	(–0.36)	0.00	(0.00–)
**Asystole**	1/32	(3.1)	0.17	(0.01–0.89)	0.06	(0.01–0.66)

Adjusted for confounding variables, including cardiac rhythm and ROSC status on hospital arrival, patient age, sex, presence of witnessed arrest, bystander CPR, bystander defibrillation, prehospital advanced airway management, and prehospital adrenaline administration.

CI, confidence interval; CPR, cardiopulmonary resuscitation; ECPR, extracorporeal cardiopulmonary resuscitation; OHCA, out-of-hospitalcardiac arrest; OR, odds ratio; PEA, pulseless electrical activity; and ROSC, return of spontaneous circulation.
