# Supplementary material for: Association of hospital-arrival rhythm and ROSC with outcomes after ECPR for OHCA with initial shockable rhythm
Source: Crit Care. 2025 Nov 3;29:466. doi: 10.1186/s13054-025-05698-9 (PMC12581255; doi:10.1186/s13054-025-05698-9)
Supplement: Supplementary file 2 — Supplementary Material 2 [file 13054_2025_5698_MOESM2_ESM.docx]

**Supplemental Methods**

**Study Design and Setting**

This study was a secondary analysis of data extracted from the JAAM-OHCA (Japanese Association for Acute Medicine Out-of-Hospital Cardiac Arrest) registry (1). The JAAM-OHCA registry is a nationwide, multicenter, prospective observational study that collects prehospital and in-hospital data from 99 medical institutions across Japan. These institutions include emergency departments and critical care medical centers certified by the Japanese Ministry of Health, Labor, and Welfare, all of which are equipped to manage critically ill patients, including those with OHCA. The study protocol was approved by the ethics committee of the Japanese Red Cross Society Kyoto Daini Hospital (Approval ID: S29-32) and the participating hospitals. The study was conducted in accordance with the Declaration of Helsinki and the requirement for informed consent was waived.

**Study Population**

We enrolled adult patients (aged ≥18 years) with OHCA due to medical causes who presented with initial shockable rhythm on emergency medical service (EMS) contact and subsequently received ECPR between June 2014 and December 2021 and were registered in the JAAM-OHCA registry database. “Medical causes” were defined as causes of arrest, excluding external factors such as trauma. Patients were excluded if they declined registry participation, either personally or through family members, or had missing data required for analysis.

**Data Collection**

Prehospital resuscitation data were sourced from the All-Japan Utstein Registry of the Fire and Disaster Management Agency (1). The “initial cardiac rhythm on EMS contact” was defined as the first observed rhythm upon the attachment of a monitor or defibrillator to a patient. The JAAM-OHCA Registry collects additional post-hospital arrival data, including baseline patient characteristics, laboratory findings, treatment specifics, causes of arrest, and outcomes. “Cardiac rhythm upon hospital arrival” indicated the initial monitored rhythm on arrival. For outcome assessment, 1-month survival and neurological status were prospectively recorded, with favorable neurological status defined as a Glasgow–Pittsburgh Cerebral Performance Category of 1 or 2.

**Cardiac Rhythm and Return of Spontaneous Circulation (ROSC) Status upon Hospital Arrival**

The primary factor of interest was cardiac rhythm upon hospital arrival. Patients were categorized into four groups based on their condition upon hospital arrival: return of spontaneous circulation (ROSC), shockable rhythm, pulseless electrical activity (PEA), and asystole. ROSC status was included in the analysis because it is clinically important to evaluate the prognosis of patients who achieved ROSC upon hospital arrival but subsequently experienced cardiac re-arrest and received ECPR.

**Outcome Measures**

The primary outcome was favorable neurological outcomes at one month. The secondary outcome was survival for at least one month.

**Sample Size Estimation**

This observational study did not include prior sample size calculations due to the retrospective nature of the analysis.

**Statistical Analysis**

Patient characteristics are presented as medians with interquartile ranges (IQRs) for continuous variables and as numbers with percentages for categorical variables. To identify potential confounders, we conducted a comprehensive review of previous studies (2-6). Based on this review, we a priori selected variables likely to influence the association between the exposure variables (cardiac rhythm and ROSC status) and outcomes. These included patient age, sex, witnessed arrest, bystander CPR, bystander defibrillation, prehospital advanced airway management, prehospital adrenaline administration, and duration from emergency call to hospital arrival (call-to-hospital interval). Age was categorized into three groups based on quartiles: 18–59, 60–69, and ≥70 years. Similarly, the call-to-hospital interval was classified into four groups based on quartiles: 0–20, 21–30, 31–40, and ≥41 minutes, with the time intervals adjusted to round numbers for easier clinical application. Crude and adjusted odds ratios (aORs) for favorable neurological and survival outcomes at one month were calculated using multiple logistic regression after adjusting for covariates. To evaluate heterogeneity, subgroup analyses were conducted based on age and call-to-hospital interval, with both variables categorized using the same classifications as those in the main analysis. Statistical analyses were performed using the R software version 4.4.2 (R Foundation for Statistical Computing). All estimates are calculated using 95% confidence intervals (CI). Missing data were not replaced or estimated.

**List of abbreviations**

aORs, adjusted odds ratios

CI, confidence intervals

ECPR, extracorporeal cardiopulmonary resuscitation

EMS, emergency medical service

IQRs, interquartile ranges

OHCA, out-of-hospital cardiac arrest

ROSC, return of spontaneous circulation

**References**

1. Kitamura T, Iwami T, Atsumi T, Endo T, Kanna T, Kuroda Y, Sakurai A, Tasaki O, Tahara Y, Tsuruta R, et al. The profile of Japanese Association for Acute Medicine - out-of-hospital cardiac arrest registry in 2014-2015. *Acute Med Surg*. 2018;5(3):249-58.

2. Kandori K, Okada Y, Okada A, Nakajima S, Okada N, Matsuyama T, Kitamura T, Narumiya H, Iizuka, R. Association between cardiac rhythm conversion and neurological outcome among cardiac arrest patients with initial shockable rhythm: a nationwide prospective study in Japan. *Eur Heart J Acute Cardiovasc Care*. 2021;10(2):119-26.

3. Rob D, Farkasovska K, Kavalkova P, Dusík M, Havranek S, Pudil J, Mockova E, Macoun J, Belohlavek J. Heart rhythm at hospital admission: A factor for survival and neurological outcome among ECPR recipients? *Resuscitation.* 2024;204:110412.

4. Alenazi A, Aljanoubi M, Yeung J, Madan J, Johnson S, Couper K. Variability in patient selection criteria across extracorporeal cardiopulmonary resuscitation (ECPR) systems: A systematic review. *Resuscitation.* 2024;204:110403.

5. Tran A, Rochwerg B, Fan E, Belohlavek J, Suverein MM, Poll MCGV, Lorusso R, Price S, Yannopoulos D, MacLaren G, et al. Prognostic factors associated with favourable functional outcome among adult patients requiring extracorporeal cardiopulmonary resuscitation for out-of-hospital cardiac arrest: A systematic review and meta-analysis. *Resuscitation.* 2023;193:110004.

6. Awad EM, Humphries KH, Grunau BE, Norris CM, Christenson JM. Predictors of neurological outcome after out-of-hospital cardiac arrest: sex-based analysis: do males derive greater benefit from hypothermia management than females? *Int J Emerg Med.* 2022;15(1):43.
