# Supplementary material for: Association of hospital-arrival rhythm and ROSC with outcomes after ECPR for OHCA with initial shockable rhythm
Source: Crit Care. 2025 Nov 3;29:466. doi: 10.1186/s13054-025-05698-9 (PMC12581255; doi:10.1186/s13054-025-05698-9)
Supplement: Supplementary file 3 — Supplementary Material 3 [file 13054_2025_5698_MOESM3_ESM.docx]

**Supplemental Limitations**

This study had some limitations. First, there is a risk of measurement bias, including potential inaccuracies in cardiac rhythm assessment and the recorded timing of key events (e.g., the moment of witnessing cardiac arrest and hospital arrival). Second, unmeasured confounders may have influenced the results; for example, we lacked data on chest compression interruption time, use of mechanical chest compressors during transport, and timing and frequency of shocks delivered by bystanders or EMS. Third, we did not evaluate rhythm changes during transport or capture the precise timing and specific rhythm at the time of in-hospital re-arrest after ROSC, limiting our ability to fully assess their impact on outcomes. Fourth, as this was a hospital-based study, a selection bias could not be ruled out. Fifth, this study has a potential bias associated with the exclusion of patients due to missing prehospital information. While most excluded patients were also ineligible for other reasons, 435 were excluded solely because of missing prehospital data. Although it remains possible that excluding these patients may have influenced the findings, this missingness appears to be at random (MAR), so excluding these cases is unlikely to have introduced substantial bias. Finally, the generalizability of our findings to settings outside of Japan remains uncertain. Future studies are needed to address these limitations and validate our findings in diverse clinical environments.
